# Spectral signature mapping and deep learning detection of Frankincense (Boswellia Sacra) using worldview-3 imagery

**DOI:** 10.1371/journal.pone.0356056

**Published:** 2026-08-13

**Authors:** Yaseen Al-Mulla

**Affiliations:** 1 Department of Soils, Water, and Agricultural Engineering, Sultan Qaboos University, Al–Khod, Oman; 2 Remote Sensing and GIS Research Center, Sultan Qaboos University, Al–Khod, Oman; RLBCAU: Rani Lakshmi Bai Central Agricultural University, INDIA

## Abstract

The identification of plant species in arid ecosystems relies on spectral signatures that capture their reflectance properties. Despite the ecological and economic importance of the Frankincense tree (Boswellia sacra), its spectral characteristics remain underexplored in remote sensing applications. This study presents a spectral characterization of Frankincense using field spectra collected with an Analytical Spectral Devices (ASD) spectroradiometer (350–2,500 nm) and validated with WorldView-3 (WV3) multispectral imagery in Oman’s Dhofar region. The ASD spectra were resampled to WV3 bands and integrated into supervised classification using the Spectral Angle Mapper (SAM) algorithm. SAM significantly outperformed NDVI thresholding, which overestimated vegetation cover and failed to distinguish Frankincense from surrounding vegetation. Accuracy assessment showed strong performance, with user’s accuracy of 88.0%, producer’s accuracy of 99.2%, overall accuracy of 93.7%, and a Kappa coefficient of 0.87. Three deep learning models (SSD, YOLOv3, and RetinaNet) were also evaluated for automated Frankincense detection. SSD achieved the best performance, detecting 701 trees with a precision of 0.83 and recall of 0.93. The findings demonstrate the potential of integrating field spectroscopy, high-resolution imagery, and deep learning for monitoring ecologically important vegetation in arid environments.

## Introduction

Frankincense (Boswellia sacra), endemic to the Dhofar region of Oman, has been central to cultural traditions, spiritual practices, and global trade networks for more than two millennia. It produces a highly valued natural resin known as Luban (olibanum). Native to the Arabian Peninsula and particularly abundant in southern Oman, this species has been exploited for centuries for its aromatic resin, which is used in medicine, religious rituals, and global trade [1]. Historically, the resin has been considered as a precious resource and traded since ancient civilizations in Arabian region [2]. The use of frankincense historically existed among numerous cultures, including the Greeks, Romans, and Egyptians, as part of their religious ceremonies [3]. The frankincense trade once linked southern Arabia with Mediterranean, Indian, and East Asian civilizations, placing the Dhofar governorate in Oman at the crossroads of cultural exchange [4]. Today, Boswellia sacra remains vital to local livelihoods and international markets, supporting industries ranging from perfumery and cosmetics to alternative medicine and pharmaceuticals. Its cultural significance has also been recognized by UNESCO, which lists the Land of Frankincense in the Dhofar region of southern Oman as a World Heritage Site. Despite this importance, frankincense populations face mounting threats in Oman and across their broader distribution in the Arabian Peninsula and Horn of Africa, including overharvesting, unsustainable grazing by camels and goats, urban expansion, and climate stressors [5–8]. Decreasing rainfall and rising temperatures, which are increasingly evident in Oman, are expected to negatively affect the regeneration, survival, and population structure of Frankincense, potentially leading to long-term population decline [5,9]. Declining regeneration rates and reduced seed viability [10,11] suggest that without scientific intervention and conservation, Boswellia sacra populations may become increasingly fragmented and degraded. Monitoring their spatial extent and health is thus crucial for sustainable management and conservation. However, traditional method of counting and documenting the mature trees is labor-intensive for tree management practices.

Remote sensing has emerged as an indispensable tool for monitoring vegetation, mapping species distributions, and assessing ecosystem health across diverse environments. With advancements in high-resolution satellite imagery and hyperspectral remote sensing, researchers can now detect subtle spectral differences between plant species, enabling applications in agriculture, forestry, biodiversity conservation, and land-use planning [12,13]. Multispectral and hyperspectral techniques provide quantitative insights into vegetation characteristics, including leaf pigment content, water stress, canopy structure, and species-specific reflectance signatures [14]. Recent advances in artificial intelligence have substantially expanded the capabilities of remote sensing for vegetation mapping and environmental monitoring. In particular, convolutional neural networks (CNNs) and, more recently, Transformer-based architectures have significantly improved object detection, semantic segmentation, and feature extraction from very-high-resolution remote sensing imagery. Modern deep learning frameworks increasingly incorporate hierarchical feature fusion, multi-scale feature representation, attention mechanisms, and boundary-guided learning to enhance the delineation and classification of vegetation and landscape objects. These developments have enabled more accurate extraction of agricultural fields, vegetation boundaries, and land-cover features from complex scenes [15–19]. Collectively, these advances demonstrate the rapid evolution of AI-driven remote sensing and provide a strong foundation for developing robust species-level vegetation mapping approaches. These advances are particularly valuable in arid and semi-arid regions, where sparse vegetation, heterogeneous backgrounds, and high soil reflectance present significant challenges for accurate vegetation mapping. However, arid and hyper-arid environments pose unique challenges to remote sensing due to high soil reflectance, sparse vegetation cover, and strong atmospheric interference. Conventional vegetation indices (e.g., Normalized Difference Vegetation Index) often have limited capability to accurately separate vegetation from background soil in such environments [20]. However, very high-resolution satellites like WorldView-3, with bands spanning visible to near-infrared wavelengths, allow for improved species mapping [21]. When combined with field-measured spectral signatures acquired with portable spectrometers (e.g., Analytical Spectral Devices (ASD)), these sensors provide powerful capabilities to characterize unique vegetation such as Frankincense trees.

Despite the global cultural and economic value of the Frankincense tree (Boswellia sacra), scientific research on its spatial distribution, ecological condition, and conservation status has been limited. Current vegetation monitoring approaches in arid and semi-arid regions—often relying on vegetation indices—are insufficient to uniquely identify individual species in heterogeneous landscapes [20,22]. In regions such as Dhofar, where sparse vegetation is interspersed with bright soils and rocky substrates, the detection of Frankincense trees remains particularly challenging.

A spectral signature represents the unique reflectance characteristics of an object across different wavelengths of the electromagnetic spectrum. By characterizing the spectral response of vegetation, remote sensing enables species identification and discrimination even in heterogeneous landscapes [23]. Spectral libraries have been developed for numerous ecosystems, including tropical forests, temperate woodlands, and agricultural crops [24]. Recent advances in hyperspectral image analysis have increasingly focused on learning more discriminative spectral representations through self-supervised learning, deep feature extraction, and Transformer-based representation learning, improving both spectral reconstruction and classification performance in high-dimensional remote sensing data [25,26]. Such developments highlight the growing importance of robust spectral feature learning and reinforce the value of accurate field-measured spectral signatures as reliable reference information for species-level vegetation discrimination and remote sensing classification.

A major gap exists in studies of spectral signatures for Frankincense. While spectral libraries exist for many plant species worldwide [23,24], such resources remain limited or unavailable for Frankincense (Boswellia sacra), highlighting a key gap in current remote sensing applications. This absence has prevented the development of reliable classification models tailored to this species. Without such a reference, conservation managers and remote sensing specialists cannot distinguish Frankincense from co-occurring shrubs and trees, nor can they track its health or population trends from satellite data. This is particularly important in light of the increasing availability of high-resolution satellite imagery optimized for vegetation studies [21,27], which offers new opportunities for species-level detection.

Although recent CNN-, Transformer-, and feature-fusion-based frameworks have significantly advanced object detection, semantic segmentation, and vegetation mapping from high-resolution remote sensing imagery, their application has largely focused on agricultural fields, land-cover classification, and generic vegetation types [15,18,19]. Species-level detection of ecologically important trees in heterogeneous arid environments remains comparatively underexplored, particularly where spectrally similar vegetation coexists with highly reflective soil and rocky backgrounds [21,27]. This gap provides an opportunity to integrate field-measured spectra acquired using ASD spectroradiometers with spectral classification and deep learning approaches for species-level detection. While vegetation indices such as the Normalized Difference Vegetation Index (NDVI) remain effective for broad vegetation monitoring, they are limited in their ability to distinguish individual species in heterogeneous arid landscapes [20]. In this context, linking an empirically validated Frankincense spectral signature with very-high-resolution WorldView-3 imagery offers a promising approach for improving species-level mapping and supporting conservation monitoring.

Beyond its ecological importance, Boswellia sacra represents an important component of Oman’s cultural heritage and regional economy through the frankincense trade. However, increasing pressures from overexploitation, grazing, and climate change highlight the need for scalable monitoring approaches that can support conservation planning and sustainable management of Frankincense populations in the Dhofar region.

The main goal of this study was to evaluate the potential of integrating field-measured spectral signatures, very-high-resolution satellite imagery, and deep learning approaches for detecting and mapping Boswellia sacra (Frankincense) in arid environments. The following specific objectives were pursued: 1) Derive a frankincense spectral signature from field spectrometer after rigorous preprocessing including despiking, smoothing, trimming, continuum removal, and normalization, 2) Align the derived spectral signature with the spectral response functions of the WorldView‑3 multispectral bands, 3) Implement, generate and analyze Spectral Angle Mapper (SAM) algorithm for classification of WV3 reflectance imagery using the reference signature and determining optimal thresholds for species‑level detection, 4) Evaluate three deep learning models namely: Single Shot Detector (SSD), You Only Look Once (YOLOv3), and RetinaNet (RN) for detecting the Frankincense, 5) Compare SAM-based Frankincense detection with NDVI-derived vegetation maps to evaluate over- and under-estimation in mixed arid environments, focusing on spectral classification approaches, 6) Conduct a statistically robust accuracy assessment using independent ground‑truth points/polygons to compute user’s accuracy (UA), producer’s accuracy (PA), overall accuracy (OA), and Cohen’s κ, and 7) Quantify classification uncertainty by calculating Wilson 95% confidence intervals for UA and PA, and by applying bootstrap resampling to derive area‑weighted OA confidence intervals.

## Materials and methods

### Study area

**Fig 1:**
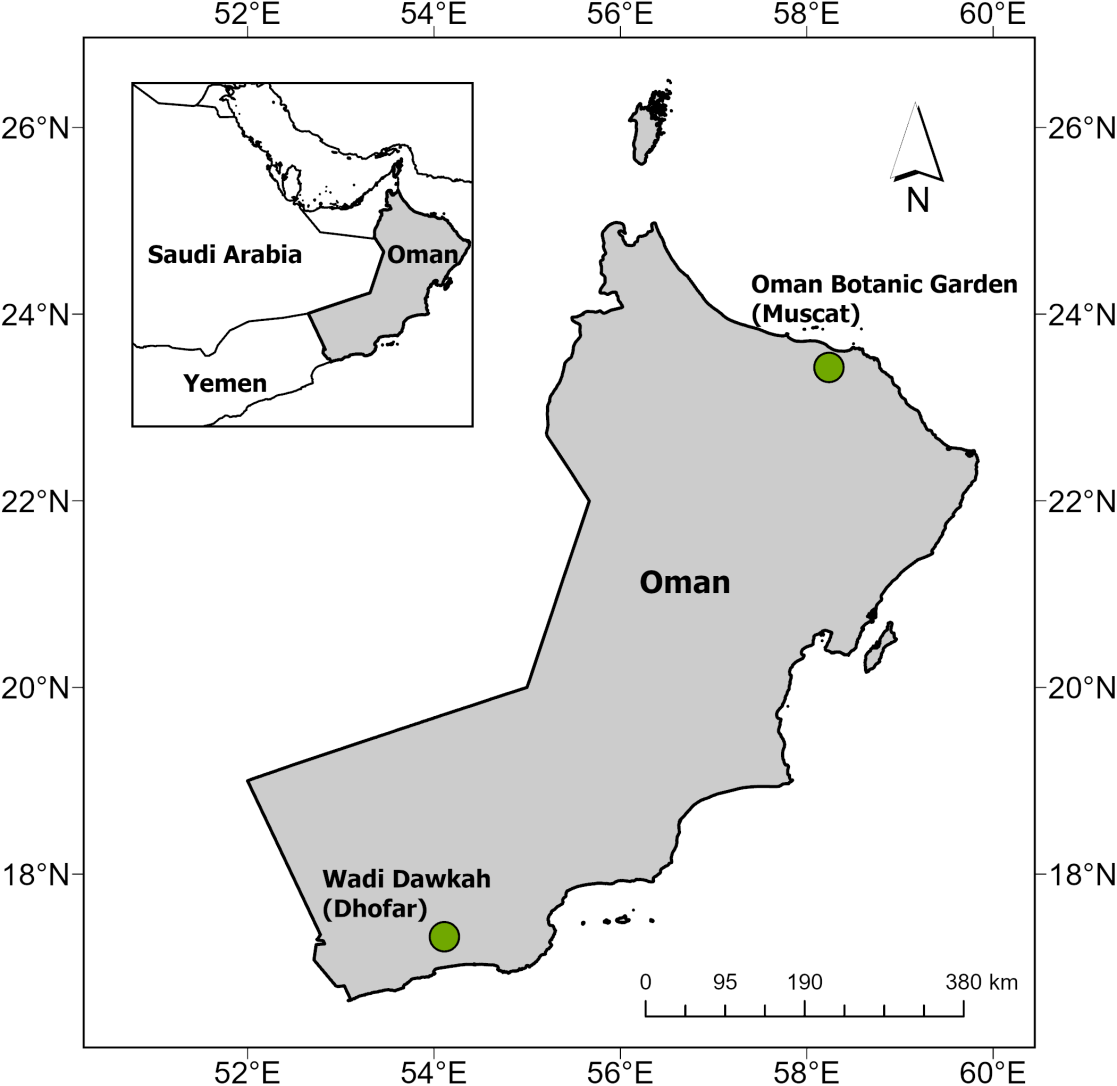
Location of the two Boswellia sacra study sites in Oman. The Oman Botanic Garden (Muscat Governorate) and Wadi Dawkah (Dhofar Governorate) are indicated by green circles. The inset map shows the location of Oman within the Arabian Peninsula. The figure was created by the authors in ArcGIS Pro using openly licensed geographic boundary data from geoBoundaries (regional inset) and the Oman Common Operational Dataset (COD-AB-OMN) distributed through the Humanitarian Data Exchange (HDX).

The study was conducted on two main locations in Oman (Fig 1): Firstly, on a preserved botanic garden large-scale project near Muscat, Oman (23.558586° N, 58.129734° E, Fig 1), which is considered as one of the world's largest botanic gardens, housing Oman's diverse plants in recreated natural habitats, from arid deserts to monsoon forests. Although these habitats are managed, the sampled Frankincense trees retain natural morphological and physiological characteristics. The spectral measurements primarily reflect intrinsic biochemical properties of the species, although some variability related to environmental conditions cannot be entirely excluded. In this location, spectral signatures of the Frankincense (Boswellia sacra) trees were determined using Analytical Spectral Devices (ASD), as described in the next sections. Secondly, on Wadi Dawkah which is a protected ancient frankincense forest in Oman's Dhofar region (17.336985° N, 54.076180° E, Fig 1), recognized as a UNESCO World Heritage Site called the “Land of Frankincense” in Dhofar Governorate of Oman [28], where the Frankincense (Boswellia sacra) trees naturally occur on limestone escarpments and wadis. Very high-resolution satellite imageries were acquired for this region to validate the determined spectral signatures of the Frankincense (Boswellia sacra) trees. Dhofar region is characterized by a semi-arid climate with a monsoon (Khareef) season from June to September, bringing unique greening compared to the rest of the Arabian Peninsula. Elevations range from 200 to 900 m a.s.l., with vegetation patches dominated by shrubs, grass, and scattered trees including Acacia, Ziziphus, and Frankincense. These management practices may influence vegetation vigor and canopy structure, potentially affecting reflectance magnitude. However, the spectral features exploited in this study, particularly those related to red-edge behavior and SWIR absorption, are primarily governed by intrinsic biochemical properties and are therefore expected to remain relatively stable across varying environmental conditions.

### Methods

**Fig 2:**
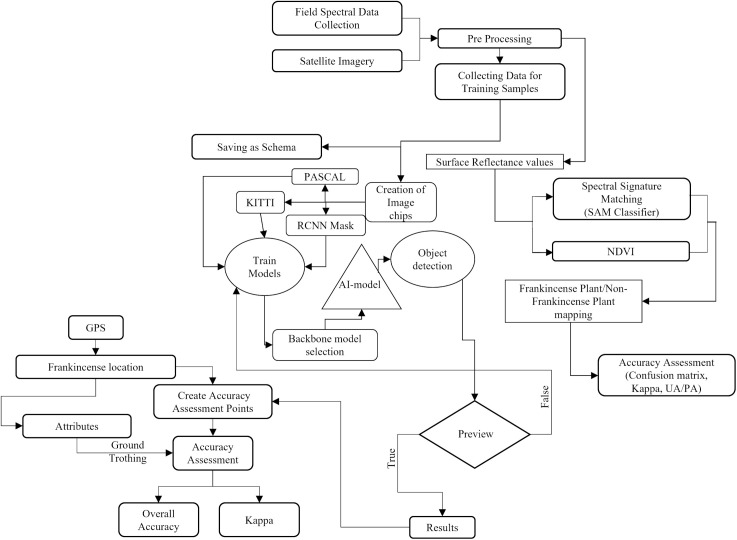
Overall layout of the study.

The following sections describe the methodology of this study for mapping Frankincense trees, while Fig 2 illustrates the overall methodology of work.

### Field spectral data collection

A FieldSpec ASD spectroradiometer (Analytical Spectral Devices Inc., Boulder, USA) was used to capture reflectance spectra of Frankincense trees. The instrument covers 350–2500 nm with spectral resolutions of 3 nm (VNIR) and 10 nm (SWIR). Field spectral measurements were conducted at the Oman Botanic Garden site near Muscat, where mature Frankincense (Boswellia sacra) trees were accessible for controlled spectroradiometer observations. A total of five Frankincense trees were sampled for spectral analysis. Spectral measurements were collected from leaves located in the upper and middle portions of the canopy to approximate the reflectance characteristics observed by satellite sensors. Measurements were conducted during cloud-free conditions in February 2019 to minimize atmospheric water vapor interference. For each sampled tree, 10 replicate measurements were collected from representative leaves using a fiber optic probe with a 25° foreoptic. A Spectralon white reference panel was used for calibration every 10 minutes. Raw ASD spectra were averaged and smoothed using a Savitzky–Golay filter to reduce noise and generate a representative spectral signature for each tree. Reflectance values were then resampled to match WorldView-3 spectral bands using spectral response functions (SRFs), and the resulting mean spectral signature was used for satellite-based classification.

### Satellite data

WorldView-3 imagery was acquired for both the Oman Botanic Garden site near Muscat and the Wadi Dawkah site in the Dhofar region in 2019. The multispectral sensor provides 8 bands (coastal, blue, green, yellow, red, red-edge, NIR1, NIR2) Table 1. It is characterized by ultra-high-resolution imagery (down to 31 cm), super-spectral capabilities with extensive visible, near-infrared, and short-wave infrared bands for detailed material analysis [29,30]. Satellite data preprocessing was performed using ENVI 5.0 and ArcGIS Pro 3.5.2 and included the following steps: (1) Radiometric correction, where digital numbers (DNs) were converted to Top-of-Atmosphere (TOA) reflectance using metadata scale factors following Kuester [31]; (2) Atmospheric correction, where the Quick Atmospheric Correction (QUAC) model was applied to derive surface reflectance. QUAC was selected because it is an image-based atmospheric correction method that does not require detailed atmospheric information at the time of image acquisition, unlike physically based methods such as FLAASH. This makes QUAC particularly suitable when ancillary atmospheric measurements are unavailable while still preserving the spectral characteristics required for vegetation classification and spectral matching [32,33]; (3) Geometric correction, where images were co-registered to ground control points with a root mean square error (RMSE) of less than one pixel; and (4) Subsetting, where the study area was extracted for analysis.

**Table 1:** Worldview 3 satellite specifications.

Spectral range	Wavelength Range (nm)	Spatial Resolution (m)
Panchromatic Band:		
Pan	450 - 800 nm	0.31
Multispectral Bands:		
Coastal Blue	400 - 450 nm	1.24
Blue	450 - 510 nm	1.24
Green	510 - 580 nm	1.24
Yellow	585 - 625 nm	1.24
Red	630 - 690 nm	1.24
Red edge	705 - 745 nm	1.24
Near-IR1	770 - 895 nm	1.24
Near-IR2	860 - 1040 nm	1.24
Feature	Specifications	
Altitude	617 km	
Swath Width	13.1 km at nadir	
Dynamic Range	11-bit Pan/MS	

### Spectral angle mapper (SAM)

The Spectral Angle Mapper (SAM) algorithm was implemented to classify each pixel in the WV3 reflectance cube against a reference Frankincense spectrum derived from ASD field spectroradiometer measurements and resampled to match WorldView-3 spectral bands. The algorithm computes the angle θ (equation 1) between the pixel spectrum and the reference in n‑dimensional space, where smaller angles indicate greater similarity [34]. Rule images were generated to guide threshold selection.


$$\begin{array}{c} \begin{array}{c} \theta ={cos}^{-\text{1}}\left(\frac{\sum_{i=\text{1}}^{n}r_{i}s_{i}}{\sqrt{\sum_{i=\text{1}}^{n}r_{i}^{\text{2}}}.\sqrt{\sum_{i=\text{1}}^{n}s_{i}^{\text{2}}}}\right) \end{array} \end{array}$$ (1)

Where $r_{i}$ is reflectance of image pixel at band i, and $s_{i}$ is reflectance of reference spectrum at band i.

### Normalized difference vegetation index (NDVI)

The Normalized Difference Vegetation Index (NDVI) is a remote sensing index that quantifies vegetation greenness and density by analyzing the light reflected and absorbed by plants. It is used to monitor plant health, assess changes in vegetation cover, and track plant growth from satellites and drones. NDVI is calculated on a per-pixel basis using the difference between near-infrared (NIR) and visible red (RED) light reflectance, divided by their sum [35–37].


$$\begin{array}{c} \begin{array}{c} NDVI=\frac{\left(NIR-RED\right)}{\left(NIR+RED\right)} \end{array} \end{array}$$ (2)

Where NIR is the reflectance in the near-infrared band. Healthy vegetation strongly reflects near-infrared light, and RED is the reflectance in the visible red band. The resulting NDVI value is a dimensionless number that ranges from −1.0 to +1.0. NDVI values provide an indication of vegetation presence and relative vigor in a pixel: Values near +1: Indicate dense, healthy, and leafy vegetation. Values between 0.2 and 0.8 represent increasingly dense, green vegetation, including shrublands and grasslands. Values near 0 generally corresponds to areas with bare soil, rock, or sand. Negative values typically indicate non-vegetated surfaces like water bodies or even clouds.

### Deep learning models

Deep learning models were implemented using the ArcGIS Pro v3.5 Python environment. Training data were prepared by manually delineating regions of interest (ROIs) around individual Frankincense tree crowns in the pan-sharpened WorldView-3 imagery. The annotated data were exported as 0.31 m pan-sharpened RGB image chips of two sizes (256 × 256 and 128 × 128 pixels) using the Export Training Data for Deep Learning tool in ArcGIS Pro. These image chips were used to train three object detection models: Single Shot Detector (SSD) [38], You Only Look Once version 3 (YOLOv3) [39], and RetinaNet [40]. Standard ArcGIS Pro implementations of these architectures were employed without modification of their input layers or network structures; consequently, the models were trained using conventional three-channel RGB imagery rather than the full eight-band multispectral data. The multispectral WorldView-3 imagery and ASD-derived spectral signatures were used exclusively for the Spectral Angle Mapper (SAM) classification. These models were selected as established benchmark object detection architectures widely used in remote sensing applications, enabling comparative evaluation of detection accuracy and computational efficiency in arid-environment imagery. The trained models were subsequently applied to detect individual Frankincense trees.

For each deep learning architecture, multiple training configurations were evaluated by varying key training parameters, including the number of training epochs, batch size, and image chip size, while maintaining the corresponding object detection framework. In total, 24 SSD configurations, 3 RetinaNet configurations, and 3 YOLOv3 configurations were evaluated. To facilitate interpretation and reproducibility, the evaluated configurations are identified in the Results (Fig 9) using descriptive labels that summarize their corresponding training settings. In these labels, the prefixes e and b denote the number of training epochs and batch size, respectively, while rsnt34, RSNT50, and DN53 indicate the ResNet-34, ResNet-50, and Darknet-53 backbone networks. For example, the label SSD_e700_b64_rsnt34 represents an SSD model trained for 700 epochs using a batch size of 64 with a ResNet-34 backbone.

### Single-shot detector

**Fig 3:**
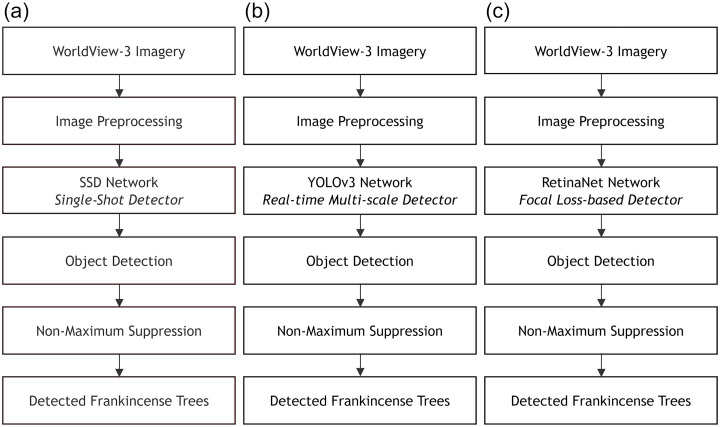
Conceptual workflows of the deep learning object detection models evaluated in this study for detecting Boswellia sacra trees from WorldView-3 imagery: (a) SSD (Single-Shot Detector), (b) YOLOv3 (Real-Time Multi-Scale Detector), and (c) RetinaNet (Focal Loss-Based Detector). Each workflow includes image preprocessing, model-specific object detection, non-maximum suppression, and generation of the final tree detections. The diagrams are original conceptual workflows created by the authors to illustrate the implementation of each model within this study.

The Single Shot Detector (SSD) (Fig 3) is a one-stage object detection model that predicts object bounding boxes and class probabilities directly from feature maps generated by a convolutional neural network backbone [38]. SSD discretizes the output space into a set of default bounding boxes with different scales and aspect ratios across multiple feature maps, enabling detection of objects at various sizes. In this study, SSD was used to detect individual Frankincense trees from high-resolution satellite imagery.

### You only look once version 3 (YOLOv3)

You Only Look Once version 3 (YOLOv3) is a real-time object detection model (Fig 3) that performs detection by dividing the input image into a grid and predicting bounding boxes and class probabilities in a single forward pass [39].

YOLOv3 uses the Darknet-53 backbone network for feature extraction and supports multi-scale detection. In this study, YOLOv3 was trained using the prepared image chips, with model performance evaluated based on precision, recall, and F1-score metrics.

### RetinaNet (RN)

RetinaNet (RN) (Fig 3) is a one-stage object detection model that combines a convolutional backbone network with a Feature Pyramid Network (FPN) to enable multi-scale object detection [40]. The FPN generates a hierarchy of feature maps from P3 (for small objects detection) to P6 (for large objects detection), allowing detection of objects at different spatial scales. The model includes two parallel subnetworks: a classification subnetwork that predicts object classes and a regression subnetwork that estimates bounding box coordinates. RetinaNet employs a focal loss function to address class imbalance during training. In this study, the RetinaNet model was applied to detect Frankincense trees and its performance was compared with SSD and YOLOv3.

### Accuracy assessment

Ground-truth data for the Dhofar study area (Wadi Dawkah) were obtained through field surveys and manual interpretation of very high-resolution satellite imagery to identify Frankincense trees and surrounding vegetation classes. Field surveys were conducted using visual identification of Frankincense trees, supported by geolocation using GPS, to ensure accurate spatial referencing of observed samples.

Ground truth polygons (Frankincense vs. Non-Frankincense) were digitized based on field observations and satellite imagery interpretation. Independent validation points were then randomly sampled within these polygons, with multiple validation points generated per polygon to ensure adequate representation of each class.

Classification accuracy was evaluated using these independent ground truth points and polygons. Following Congalton and Green [41], an error matrix was generated to compute user’s accuracy (UA), producer’s accuracy (PA), overall accuracy (OA), and Cohen’s Kappa coefficient (κ).

Wilson 95% confidence intervals (CI) were calculated for UA and PA [42], while area-weighted OA was estimated using bootstrap resampling following Olofsson et al. [43].

For the evaluation of the deep learning object detection models, the ArcGIS Pro 3.5 Compute Accuracy For Object Detection tool was used. A Minimum Intersection over Union (IoU) threshold of 0.50 was adopted to determine whether a detected bounding box corresponded to a ground-truth object. Predictions with an IoU ≥ 0.50 were considered True Positives, whereas detections with lower overlap were classified as False Positives. Ground-truth objects that were not matched by any prediction satisfying this threshold were counted as False Negatives. This IoU threshold follows the standard object detection evaluation protocol implemented in ArcGIS Pro and is consistent with the widely adopted PASCAL VOC evaluation criterion

Detection precision (P), recall (R), and F1-score were also calculated to evaluate model performance [44]. In addition, model performance was further evaluated using Receiver Operating Characteristic (ROC) and Precision–Recall (PR) analyses. The ROC curve illustrates the relationship between the true positive rate (TPR) and the false positive rate (FPR) across different detection thresholds, providing an overall assessment of the model’s discriminative ability. The Precision–Recall (PR) curve evaluates the trade-off between precision and recall across varying confidence thresholds and is particularly informative for classification tasks where class imbalance may occur. These curves were generated from the outputs of the evaluated models, including both the Spectral Angle Mapper (SAM) classifier and the deep learning models (SSD, YOLOv3, and RetinaNet), to compare their performance.

The UA, PA, OA, κ, CI, P, R and F1_score parameters were calculated using the following equations:


$$\begin{array}{c} \begin{array}{c} {UA}_{i}=\frac{n_{ii}}{\sum_{j}n_{ij}} \end{array} \end{array}$$ (3)

Where ${UA}_{i}$ is user’s accuracy for class i, $n_{ii}$ is number of correctly classified samples of class i, $\sum_{j}n_{ij}$ is total number of samples classified as class i, and j is an index across all reference classes.


$$\begin{array}{c} \begin{array}{c} {PA}_{i}=\frac{n_{ii}}{\sum_{j}n_{ij}} \end{array} \end{array}$$ (4)

Where ${PA}_{i}$ is user’s producer’s accuracy for class i, $n_{ii}$ is number of correctly classified samples of class i, $\sum_{j}n_{ij}$ is total number of reference samples of class i, and j is an index across outputs.


$$\begin{array}{c} \begin{array}{c} OA=\frac{\sum_{i}^{k}n_{ii}}{N} \end{array} \end{array}$$ (5)

Where OA is overall classification accuracy, $n_{ii}$is number of correctly classified samples of class i, k is total number of classes in the classification, and N is total number of reference (ground truth) samples.


$$\begin{array}{c} \begin{array}{c} \kappa =\frac{N\cdot \sum_{i=\text{1}}^{k}n_{ii}-\sum_{i=\text{1}}^{k}\left(n_{i+}\cdot n_{+i}\right)}{N^{\text{2}}-\sum_{i=\text{1}}^{k}\left(n_{i+}\cdot n_{+i}\right)} \end{array} \end{array}$$ (6)

Where κ is Cohen’s Kappa coefficient (agreement corrected for chance), N is total number of reference (ground truth) samples, $n_{ii}$ is number of correctly classified samples in class i (diagonal element of confusion matrix), $n_{i+}$ is row sum for class i (all samples classified as class i), $n_{+i}$ is column sum for class i (all reference samples belonging to class i), and k is total number of classes.


$$\begin{array}{c} \begin{array}{c} CI=\frac{\hat{p}+\frac{z^{\text{2}}}{\text{2}n}\pm z\cdot \sqrt{\frac{\hat{p}\left(\text{1}-\hat{p}\right)}{n}+\frac{z^{\text{2}}}{\text{4}n^{\text{2}}}}}{\text{1}+\frac{z^{\text{2}}}{n}} \end{array} \end{array}$$ (7)

Where CI is confidence interval bounds (lower and upper), $\hat{p}$ is observed proportion (e.g., UA or PA value), n is number of validation samples in that class, and z is critical value from the standard normal distribution. For a 95% confidence level, z = 1.96.


$$\begin{array}{c} \begin{array}{c} P=\frac{TP}{\left(TP+FP\right)} \end{array} \end{array}$$ (8)

$$\begin{array}{c} \begin{array}{c} R=\frac{TP}{\left(TP+FN\right)} \end{array} \end{array}$$ (9)

$$\begin{array}{c} \begin{array}{c} F{\text{1}}_{S}core=\frac{\text{2}PR}{P+R} \end{array} \end{array}$$ (10)

Where FP is false positives, FN is false negatives, and TP is true positives.

## Results

### Frankincense spectral signature

**Fig 4:**
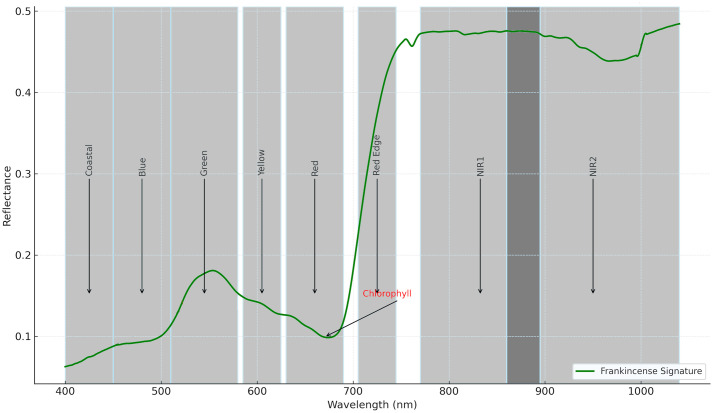
Frankincense spectral signature with annotated absorption features and worldview-3 bands. Grey areas represent worldview 3 bands, while dark grey represents overlap of NIR1 & NIR2.

The ASD field measurements yielded a distinctive spectral signature of Boswellia sacra across the visible and near-infrared (VNIR–NIR) range (Fig 4). The reflectance curve exhibited three notable features:

(1) a green peak around 550 nm, associated with chlorophyll reflectance;
(2) a red absorption trough near 670 nm due to chlorophyll absorption; and
(3) a pronounced near-infrared (NIR) plateau beyond 750 nm, typical of vegetation canopy scattering.

A clear red-edge transition between 680–740 nm was also observed, indicating strong vegetation activity.

Fig 4 also illustrates the correspondence between the ASD-derived spectral signature and the spectral bands of the WorldView-3 sensor, demonstrating that key spectral characteristics are preserved after resampling and are suitable for vegetation detection and classification.

### Satellite-based detection

**Fig 5:**
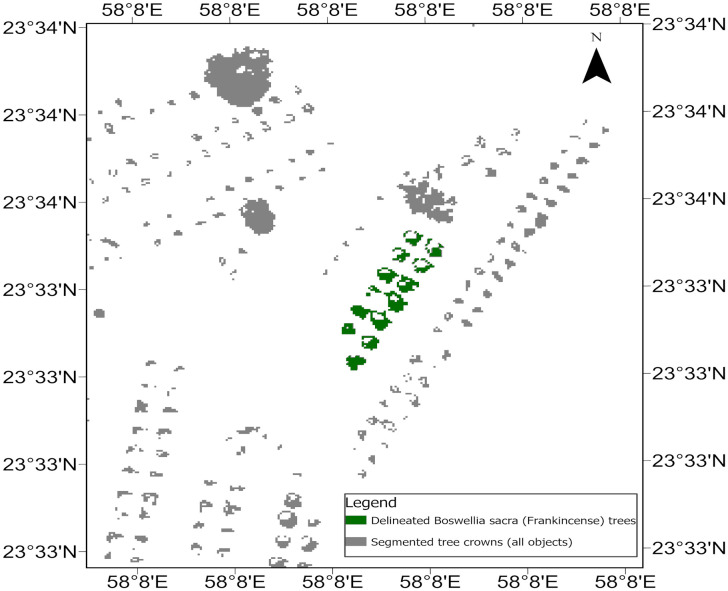
Delineation of Boswellia sacra tree crowns in the Oman Botanic Garden using the Spectral Angle Mapper (SAM) algorithm. Gray polygons represent all segmented tree crowns generated during image segmentation, whereas green polygons indicate tree crowns classified as Boswellia sacra based on the ASD-derived reference spectral signature.

**Fig 6:**
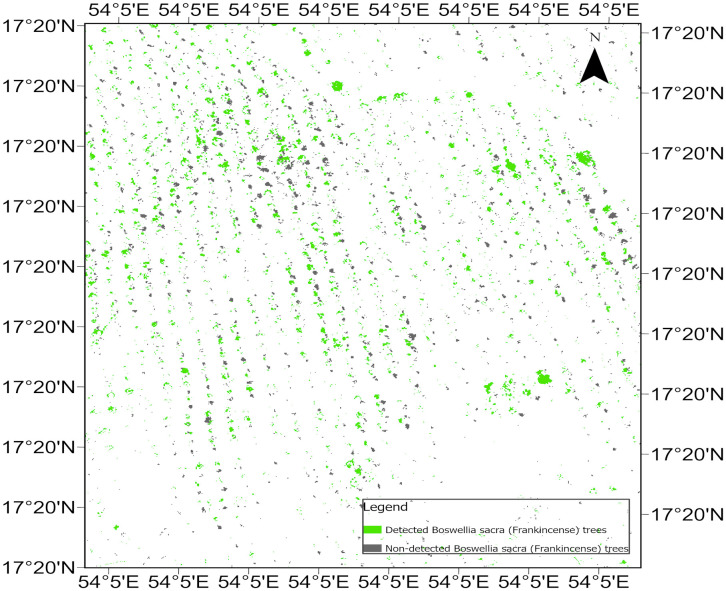
Spatial distribution of Boswellia sacra tree crowns identified by the Spectral Angle Mapper (SAM) algorithm in the Wadi Dawkah UNESCO World Heritage Site, Dhofar, Oman. Gray objects represent all segmented vegetation objects generated during image segmentation, while green objects indicate vegetation segments classified as Boswellia sacra using the ASD-derived reference spectral signature.

The Spectral Angle Mapper (SAM) algorithm successfully delineated Frankincense trees in the botanical garden site (Fig 5) and was subsequently applied to the Wadi Dawkah region in Dhofar (Fig 6). The resulting spatial patterns revealed a heterogeneous distribution of Frankincense trees, with noticeable clustering in certain areas of the study site. These spatial patterns are consistent with the known ecological behavior of Boswellia sacra, which typically occurs in patchy distributions within arid landscapes.

Classification accuracy assessment indicated strong performance, with an overall accuracy of 93.7% and a Cohen’s Kappa coefficient of 0.873, confirming the reliability of the SAM-based detection results.

**Fig 7:**
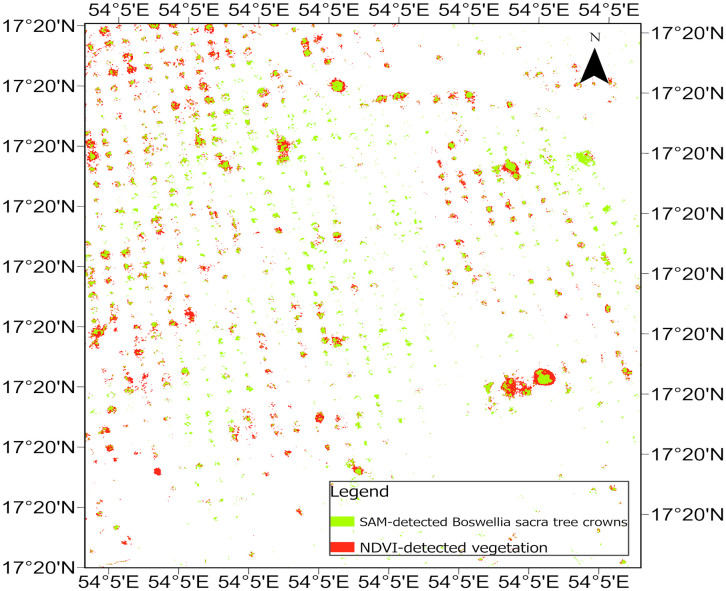
Comparison between vegetation identified using the Normalized Difference Vegetation Index (NDVI) and Boswellia sacra tree crowns identified using the Spectral Angle Mapper (SAM) algorithm in the Wadi Dawkah study area. Green objects indicate Boswellia sacra tree crowns detected by the SAM algorithm based on the ASD-derived reference spectral signature, whereas red objects represent vegetation identified using the NDVI approach. The comparison illustrates the broader vegetation extent identified by NDVI relative to the species-specific SAM classification.

To assess robustness, NDVI-based thresholding was compared with the SAM classification. The NDVI maps (Fig 7) represent general vegetation cover, whereas the SAM results specifically identify Frankincense trees. NDVI tended to overestimate vegetation presence by 39.4% relative to SAM-derived Frankincense detections within the same spatial extent. This overestimation was calculated by comparing the total area classified as vegetation by NDVI with the area identified as Frankincense by SAM within the same spatial extent.

This discrepancy reflects the inherent limitation of NDVI as a broadband vegetation index, which responds to general photosynthetic activity rather than species-specific reflectance properties. In contrast, SAM utilizes the full spectral signature derived from ASD measurements, enabling more precise identification of Frankincense. As a result, while NDVI remains effective for broad-scale vegetation mapping, it is not suitable for species discrimination in heterogeneous arid environments.

### Detection from the deep learning models

In this study, the three models (SSD, YOLOv3, and RetinaNet) were trained using the same dataset and training configuration. Model performance improved progressively with training, as indicated by a decrease in both training and validation loss over successive epochs, with no evidence of overfitting observed.

**Fig 8:**
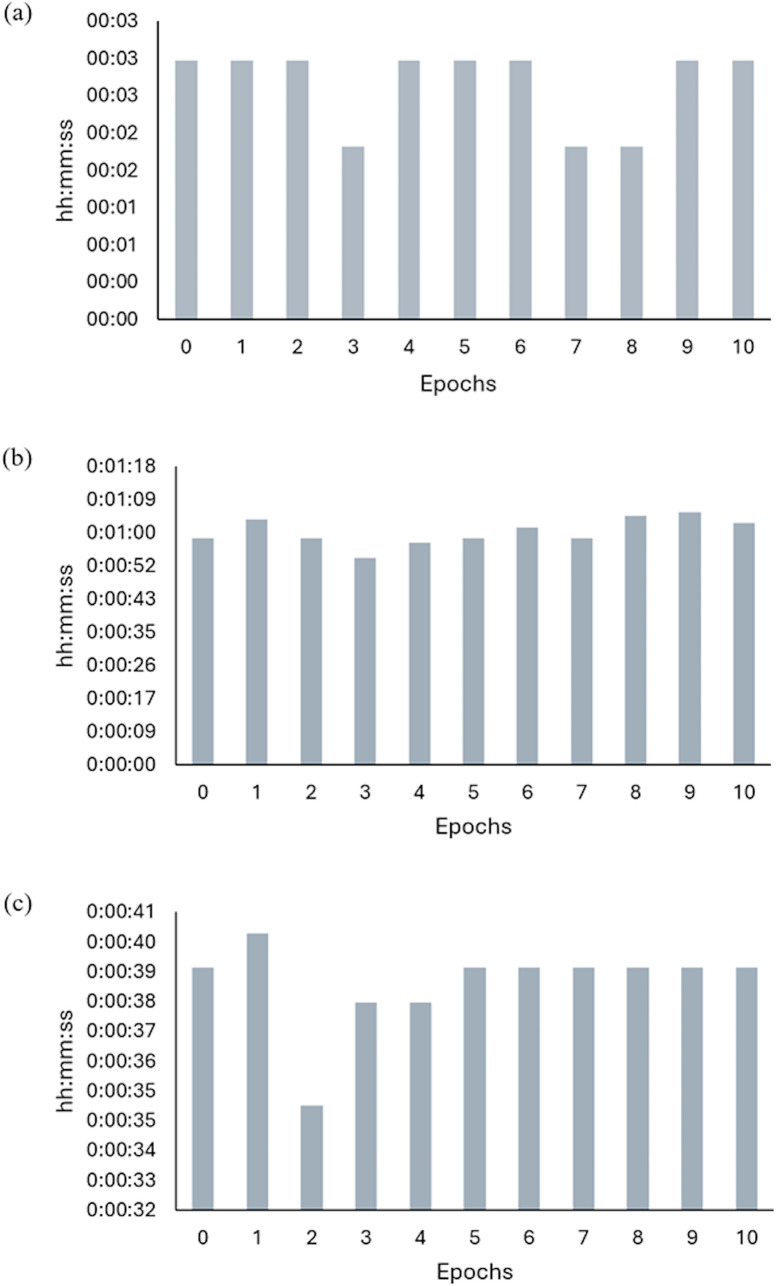
Training time per epoch for the evaluated deep learning models (SSD, YOLOv3, and RetinaNet). The figure highlights differences in computational efficiency, with SSD requiring substantially less training time per epoch compared to YOLOv3 and RetinaNet.

The computational efficiency of the models differed significantly. The average training time per epoch for each model is shown in Fig 8. The SSD model required approximately 2 seconds per epoch, whereas YOLOv3 required approximately 52 seconds per epoch. RetinaNet showed intermediate performance. Despite using the same training data, number of epochs, and computational environment, SSD demonstrated both higher computational efficiency and better detection performance compared to the other models.

**Fig 9:**
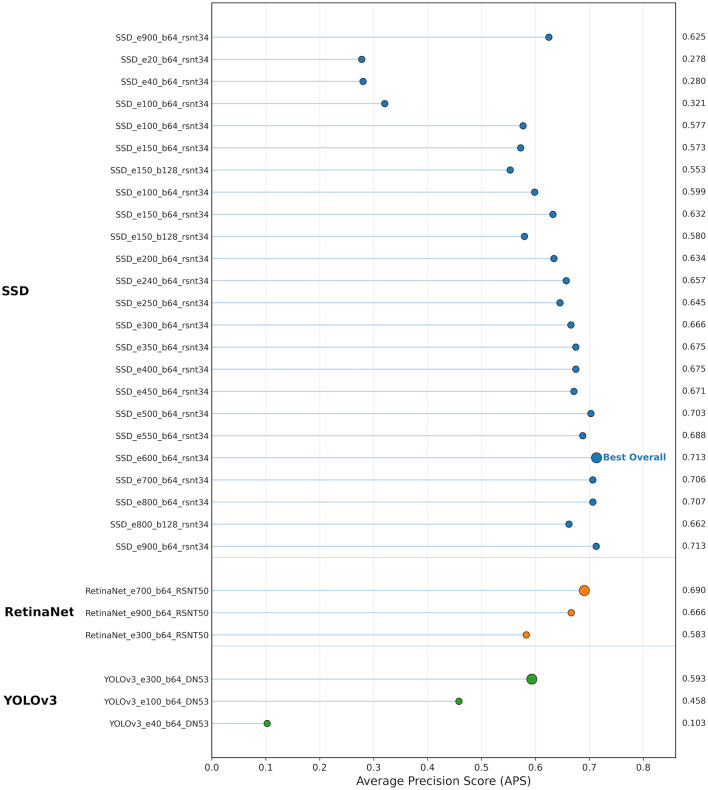
Average precision scores (APS) of the evaluated training configurations for the three deep learning architectures (SSD, RetinaNet, and YOLOv3) used for Boswellia sacra tree detection from WorldView-3 imagery. Each point represents an individual training configuration, and the enlarged point denotes the highest-performing configuration within each architecture. The annotation “Best Overall” identifies the highest-performing configuration among all evaluated models. Configuration labels summarize the principal training settings in the format Architecture_eEpochs_bBatchSize_Backbone (e.g., SSD_e700_b64_rsnt34).

The overall average precision score of all three deep learning models used in this study with different arguments and environments, using the same training data and imagery chip sizes (Fig 9) showed that the SSD model has the highest precision score of 0.713 using 600 and 900 epochs. On the other hand, the RN model recorded the second highest APS value of 0.69 by using 700 epochs, but 0.67 using 900 epochs. While the YOLOv3 model recorded an APS value of 0.59 using 300 epochs. It was observed in the case of YOLOv3 that it was not able to train above 300 epochs. The trained models in this study showed that the SSD model achieved the highest accuracy in the training.

The SSD, YOLOv3, and RetinaNet models were trained using identical datasets derived from WorldView-3 imagery. Each model was evaluated using precision, recall, and F1-score metrics. Table 2 summarizes the comparative performance of the three models.

**Table 2:** Quantitative evaluation of deep learning models performance for Frankincense tree detection.

Model	Precision	Recall	F1-score	True Positives	False Positives	False Negatives
SSD	0.83	0.93	0.87	701	148	53
YOLOv3	0.37	0.37	0.37	276	470	468
RetinaNet	0.69	0.54	0.61	403	181	341

### Accuracy assessment

**Fig 10:**
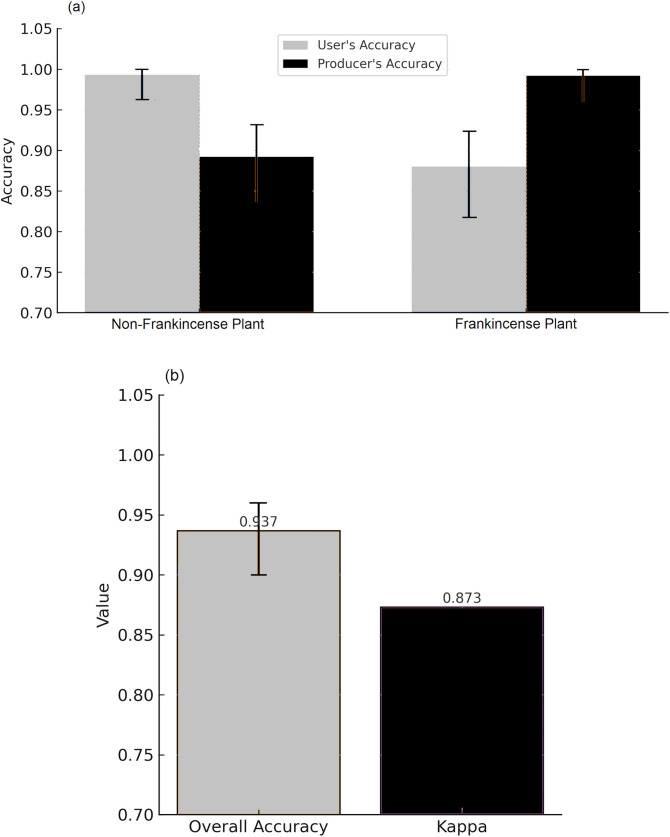
Accuracy assessment of Frankincense detection using the spectral angle mapper (SAM) classification applied to WorldView-3 imagery. Panel (a) presents the User's Accuracy (UA) and Producer's Accuracy (PA) for the two classes (Non-Frankincense and Frankincense), together with their corresponding 95% Wilson confidence intervals. Panel (b) shows the Overall Accuracy (OA) and Cohen's κ coefficient derived from the confusion matrix. Error bars represent the 95% bootstrap confidence interval associated with the Overall Accuracy, whereas Cohen's κ is presented as a summary measure of classification agreement beyond chance. Overall, the results demonstrate the high reliability and robustness of the SAM-based classification.

Validation using 300 independent ground‑truth points/polygons yielded high agreement between SAM detections and reference data. The error matrix produced an overall accuracy (OA) of 0.937, user’s accuracy (UA) of 0.880, producer’s accuracy (PA) of 0.992, and Cohen’s κ = 0.873 (Fig 10). Wilson 95% confidence intervals confirmed statistical robustness: UA (0.89–0.95), PA (0.98–0.99). Area‑weighted OA, computed via 1000 bootstrap resamples, produced a mean of 0.94 with 95% CI (0.92–0.95). These results indicate a reliable detection workflow (Fig 10). The NDVI performance produced lower accuracies than those of SAM (Table 3) where the NDVI’s error matrix produced an overall accuracy (OA) of 0.810, user’s accuracy (UA) of 0.720, producer’s accuracy (PA) of 0.845, and Cohen’s κ = 0.600.

**Table 3:** Comparative accuracy performance of SAM vs. NDVI classifications.

Metric	SAM (Spectral Angle Mapper)	NDVI (Threshold Classification)
User’s Accuracy (UA)	0.880	0.720
Producer’s Accuracy (PA)	0.992	0.845
Overall Accuracy (OA)	0.937	0.810
Kappa Statistic	0.873	0.600

**Fig 11:**
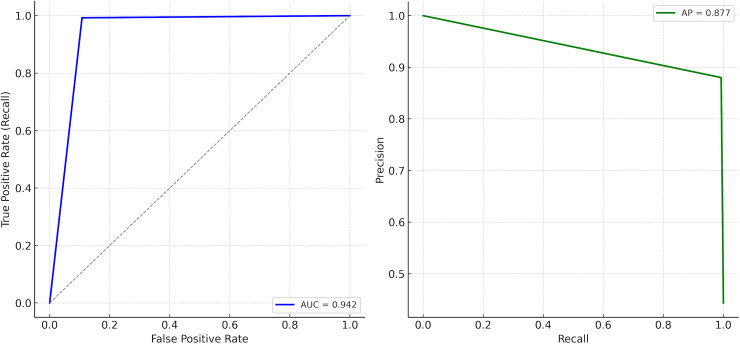
Left: Receiver operating characteristic (ROC) curve. Right: Precision–Recall (PR) curve.

The evaluation of the Spectral Angle Mapper (SAM) classifier using Receiver Operating Characteristic (ROC) and Precision–Recall (PR) curves demonstrated the robustness of the extracted Frankincense spectral signature. The ROC curve (Fig 11) shows that the classifier consistently achieved a high True Positive Rate while maintaining a low False Positive Rate, yielding an Area Under the Curve (AUC) greater than 0.90. This indicates excellent discriminative capacity for separating Frankincense trees from surrounding non-plant features in the Dhofar landscape. The Precision–Recall curve (Fig 11) highlighted that classification precision consistently exceeded 90% across recall levels with Average Precision (AP) above 0.8, indicating minimal false positives even at broader classification thresholds. These results confirm that the Frankincense spectral signature extracted from ASD spectroradiometer measurements, when integrated with WorldView-3 imagery, provided a reliable and scalable means for operational mapping of this ecologically and culturally significant species.

**Fig 12:**
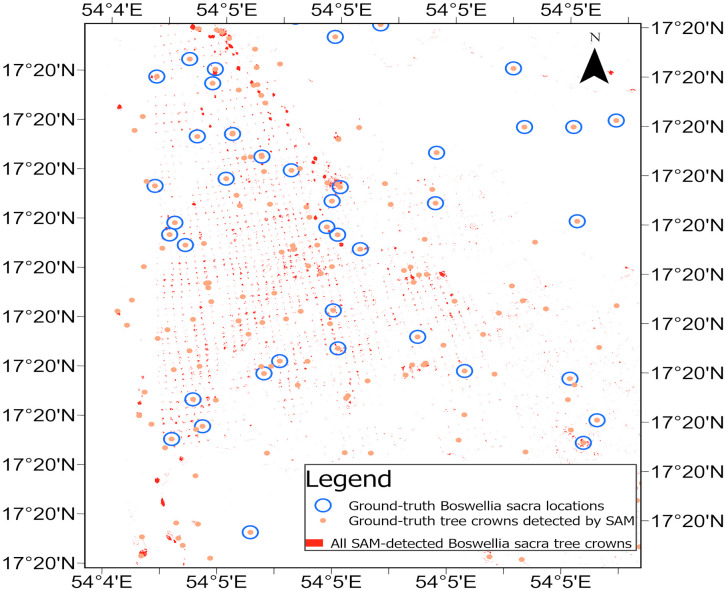
Validation of the spectral angle mapper (SAM) classification using independently collected ground-truth observations in the Wadi Dawkah study area. Red polygons represent all Boswellia sacra tree crowns detected by the SAM algorithm. Blue circles indicate independently surveyed ground-truth locations of Boswellia sacra trees, while orange points identify the subset of SAM-detected tree crowns corresponding to those ground-truth locations. The close spatial correspondence between the orange points and blue circles demonstrates the accuracy of the proposed detection approach.

For the accuracy assessment of the SSD deep learning model, a vector layer containing the GPS coordinates and tree information was constructed and overlaid over the SSD based detected Frankincense trees (Fig 12). A quantitative breakdown of detection performance, including true positives, false positives, and false negatives, is presented in Table 2. The assessment resulted in the SSD model being used in this study being able to detect 701 Frankincense trees that matched the ground truth points with a precision of 0.83 and a recall value of 0.93. The analysis also showed that the SSD based model recorded 148 as false positive Frankincense trees, while 46 trees were not detected. Most false positives were associated with spectrally or structurally similar vegetation patches and shadowed areas within the heterogeneous arid landscape. Hence, the SSD model exhibited strong performance in all areas and has considerable potential for operational monitoring applications.

## Discussion

### Spectral characteristics of Frankincense

The field ASD measurements demonstrated that Frankincense (Boswellia sacra) exhibits a distinct reflectance profile characterized by a pronounced red-edge transition and notable absorption features in the shortwave infrared region associated with lignin–cellulose content. These spectral characteristics are consistent with known vegetation reflectance behavior and provide a basis for species detection when combined with appropriate classification methods.

Previous studies have highlighted the utility of hyperspectral signatures in distinguishing desert tree species such as Acacia and Prosopis [24,45]. In this study, the derived spectral signature of Frankincense was successfully used within the SAM classification framework, demonstrating its effectiveness for identifying this species in arid environments.

It should be noted that the spectral measurements used in this study were obtained from Frankincense trees located in the Oman Botanic Garden near Muscat, while satellite-based validation was conducted in the Wadi Dawkah region of Dhofar. Management practices such as reduced interspecific competition and occasional irrigation may influence canopy density and vegetation vigor, potentially affecting reflectance intensity. However, the key spectral features used to identify Frankincense particularly the pronounced red-edge slope and SWIR absorption characteristics are primarily associated with intrinsic biochemical properties of the species and are therefore expected to remain relatively stable across different environmental conditions. Future studies incorporating additional natural populations across the broader distribution of Boswellia sacra would further strengthen the general applicability of the identified spectral signature.

### Satellite-based detection

Building on the identified spectral characteristics, satellite-based classification methods were evaluated to determine whether the Frankincense spectral signature could be reliably detected in very high-resolution imagery. The application of WorldView-3 imagery, with its 8-band multispectral configuration and high spatial resolution, proved highly effective for delineating Frankincense stands. The Spectral Angle Mapper (SAM) achieved high classification accuracies (UA = 88.0%, PA = 99.2%), comparable to or exceeding results reported for other arid vegetation mapping tasks using WV3 [46].

In contrast, NDVI-based thresholding failed to adequately discriminate Frankincense from surrounding vegetation, confirming the limitations of broadband vegetation indices for species discrimination in heterogeneous arid landscapes [47]. SAM’s success highlights the advantage of utilizing full spectral similarity when field reference spectra are available.

The high overall accuracy (93.7%) and Kappa coefficient (0.873) confirm the robustness of the classification results. Wilson 95% confidence intervals and bootstrap resampling further demonstrated model reliability and generalization capability. High classification accuracy was achieved despite the spectral similarity between Frankincense and other desert-adapted vegetation, emphasizing the importance of field-collected reference spectra for accurate species detection [23,48].

The results demonstrate that very high-resolution multispectral imagery calibrated with field spectroscopy can provide a practical approach for operational monitoring of Frankincense populations in arid regions. While hyperspectral satellites such as EnMAP and PRISMA may further improve detection performance, the present study shows that commercially available multispectral datasets already provide substantial utility for vegetation mapping applications.

Traditional field-based monitoring of Frankincense populations is often labor-intensive and spatially limited. The integration of field spectroscopy with satellite-based classification therefore provides an efficient framework for supporting vegetation monitoring and conservation planning. Nevertheless, both the training and validation sites used in this study represent relatively controlled or managed environments. Additional validation across broader natural landscapes is required to further assess the scalability and transferability of the proposed methodology.

### Comparative performance of deep learning models

In addition to spectral classification approaches, deep learning–based object detection models were evaluated to assess their ability to identify individual Frankincense trees from high-resolution imagery.

The comparative evaluation of SSD, YOLOv3, and RetinaNet highlights the trade-offs between detection accuracy and computational efficiency. Among the evaluated models, SSD demonstrated the strongest overall performance, achieving the highest precision score together with strong recall while requiring substantially less training time per epoch than YOLOv3 and RetinaNet. These findings are consistent with previous studies reporting SSD as a lightweight and computationally efficient alternative for object detection tasks involving high-resolution imagery [38,49].

The relatively lower performance of YOLOv3 and RetinaNet suggests that more computationally complex architectures do not necessarily yield improved results for single-species detection tasks in arid environments. The inability of YOLOv3 to scale beyond 300 epochs under the current training configuration further highlights the challenges associated with heavier detection architectures when computational resources are limited.

Recent advances in remote sensing have introduced increasingly sophisticated deep learning frameworks that integrate Transformer modules, hierarchical feature fusion, multi-scale feature representation, and boundary-guided learning to improve object detection and semantic segmentation in complex landscapes [15,18,19]. These methods have demonstrated remarkable performance for applications such as cropland mapping and large-scale land-cover extraction. However, many of these architectures have been developed and evaluated using extensive annotated datasets and relatively homogeneous target classes. In contrast, the present study focuses on species-level detection of individual Frankincense trees within heterogeneous arid environments characterized by sparse vegetation, bright soil backgrounds, and limited training samples. Under these conditions, the SSD architecture achieved the best balance between detection accuracy and computational efficiency, indicating that well-established object detection frameworks remain highly effective for targeted ecological monitoring where computational simplicity, robustness, and practical implementation are important considerations

Overall, the results demonstrate that integrating field-measured spectral signatures with very-high-resolution WorldView-3 imagery and deep learning provides an effective framework for species-level detection of Frankincense trees in heterogeneous arid environments. The superior balance achieved by the SSD model between detection accuracy and computational efficiency suggests that well-established object detection architectures remain practical and reliable solutions for ecological monitoring applications where training data and computational resources may be limited. These findings highlight the value of combining spectral characterization with deep learning to support biodiversity assessment, conservation planning, and long-term monitoring of ecologically and culturally important tree species. Future research should investigate larger and more diverse annotated datasets, multi-temporal and multi-season imagery, additional ecological conditions, and emerging deep learning architectures to further improve detection accuracy, robustness, and generalization across different arid landscapes.

## Conclusion

This study demonstrated the potential of integrating field spectroscopy, high-resolution satellite imagery, and deep learning approaches for detecting and mapping Boswellia sacra in arid environments. The results show that the Spectral Angle Mapper (SAM) classification using WorldView-3 imagery can effectively distinguish Frankincense trees within the study area, achieving an overall accuracy of 93.7% with a Kappa coefficient of 0.87. In comparison with NDVI-based vegetation mapping, the SAM approach provided improved species discrimination in heterogeneous arid landscapes.

The deep learning models further enhanced detection capabilities for identifying individual Frankincense trees from high-resolution imagery. Among the evaluated models, the Single Shot Detector (SSD) demonstrated the best performance, achieving a precision of 0.83 and recall of 0.93, indicating strong potential for automated monitoring of Frankincense populations.

Despite these promising results, several limitations should be acknowledged. The spectral measurements and training datasets were derived from a limited number of sites, which may not fully represent the ecological variability of Boswellia sacra across its broader geographic distribution. In addition, the analysis relied on multispectral satellite imagery, which provides lower spectral resolution than hyperspectral sensors. Future research should expand field sampling across multiple ecological regions and explore the integration of hyperspectral and UAV-based observations to further improve species detection and support long-term conservation and management of Frankincense ecosystems.

The proposed framework may support environmental authorities and local stakeholders in Oman by enabling more efficient monitoring of Frankincense populations and contributing to conservation planning and sustainable management of this culturally and economically important species.

## Supporting information

S1 File Boswellia spectral dataset. (XLSX)
